# Editorial: Roles of granzymes in inflammation, aging, and autoimmunity

**DOI:** 10.3389/fimmu.2025.1672898

**Published:** 2025-08-11

**Authors:** Fehim Esen, Christopher T. Turner

**Affiliations:** ^1^ Department of Ophthalmology, Cornea, Uveitis, and Ocular Immunology Sections, Istanbul Medeniyet University School of Medicine, Istanbul, Türkiye; ^2^ Future Industries Institute, University of South Australia, Adelaide, SA, Australia

**Keywords:** granzyme, immune-secreted protease, chronic disease, inflammation, autoimmunity, cytotoxic, therapeutic target

Granzymes are a family of immune-secreted proteases traditionally identified as having roles triggering cell death in virally infected and cancerous cells. In recent years our view of granzymes has evolved and we now recognize granzymes to have additional non-cytotoxic roles. Granzymes are elevated in response to tissue inflammation and have become the focus of interest as they contribute to disease pathogenesis, with negative roles identified in extracellular matrix remodeling (1–3), inflammation (4–6), wound healing (7–9), autoimmunity (10, 11), and aging (12, 13). As such, granzymes are emerging as potential therapeutic targets for a range of diseases characterized by tissue injury and inflammation. Saliently, the five granzymes have distinct roles, a fact increasingly supported through numerous emerging studies including those centered on the ‘orphan’ granzymes (i.e., granzyme K), which display newly uncovered mechanistic roles and substrate specificities. The Research Topic “Roles of granzymes in inflammation, aging, and autoimmunity” in Frontiers in Immunology is therefore timely, as it captures a snapshot of this exciting research area. Below are summaries of those original research studies and literature reviews selected for publication in this Research Topic.

Aybay et al. published a study called “Extended cleavage specificities of human granzymes A and K, two closely related enzymes with conserved but still poorly defined functions in T and NK cell-mediated immunity”. Using phage display analysis, the authors investigated the cleavage specificities of two distinct human granzymes—granzyme A and granzyme K—which originate from separate branches of the phylogenetic tree. While both enzymes demonstrated similar selectivity at the P1 position, significant differences were observed in the N-terminal region of the cleavage site. Notably, the authors raised concerns about earlier findings regarding granzyme A–induced cytokine and chemokine expression, showing that recombinant granzyme A preparations were contaminated with trace amounts of lipopolysaccharide (LPS), which alone were sufficient to induce inflammatory cytokine and chemokine production. A more comprehensive understanding of the cleavage preferences of granzymes A and K is crucial for the precise identification of their potential binding targets and for elucidating their still poorly defined roles in vertebrate biology.

Cigalotto and Martinvalet published a review titled “Granzymes in health and diseases: the good, the bad and the ugly” (15). The authors summarized recent advances in understanding the diverse physiological and pathological roles of granzymes, which were once considered merely cytotoxic mediators. In addition to their intracellular functions, granzymes have been shown to exert various extracellular effects, including extracellular matrix remodeling, vascularization, cell migration, alteration of surface receptors and cellular functions. While these extracellular activities are essential for maintaining tissue homeostasis, in certain clinical contexts—particularly chronic inflammation and autoimmunity—they can also lead to excessive matrix degradation and cell death. Additional roles of granzymes have been implicated in tumor immunology and invasion, aging, angiogenesis, wound healing, and fibrosis.

Gill et al. published a review called “Exploring the role of granzyme B in subretinal fibrosis of age-related macular degeneration”. This review emerges from multiple recent published articles by senior author Matsubara identifying role/s for granzyme B in age-related macular degeneration (AMD) (2, 14, 15). As a major cause of blindness in elderly individuals in developed countries, AMD requires detailed investigation to identify new approaches for disease treatment. The authors highlighted choroidal neovascularization and the subsequent development of subretinal fibrosis as critical contributors to disease progression, leading to subretinal scarring and damage to the choriocapillaris, retinal pigment epithelium, and photoreceptors. Mast cells containing granzyme B are elevated in association with elevated inflammation that is a hallmark of AMD. Detailed is the emerging role of GzmB in both choroidal neovascularization and subretinal fibrosis and an exploration of the potential of GzmB-related therapeutic targets for inhibiting subretinal fibrosis in AMD. Although various immunological pathways have been implicated in neovascular AMD, current treatments rely solely on anti-angiogenic monoclonal antibodies, which do not address subretinal fibrosis. Granzyme B thus emerges as a promising immunologic target to modulate the immunopathogenesis of AMD and prevent both neovascularization and fibrotic progression.

Aubert et al. presented a skin focused study entitled “Potential implications of granzyme B in keloids and hypertrophic scars through extracellular matrix remodeling and latent TGF-β activation”. Keloid and hypertrophic scars involve ‘excess’ wound healing, with the scar tissue extending beyond the injury border. Caused in response to excess fibrotic activity, granzyme B was identified as contributing to disease development. Granzyme B was elevated in keloids and hypertrophic scars, localizing within the dermis and primarily expressed by mast cells. Mechanistically, granzyme B functioned through augmented extracellular matrix remodeling and the cleavage of extracellular matrix molecules involved in the regulation of latent TGF-β activation.

Turner, one of the authors of this Editorial, presented a minireview called “Pro-inflammatory granzyme K contributes extracellularly to disease”. Granzyme K is identified as an orphan granzyme as the amount of published data on it has, until recently, been minimal. However, there has been an increased interest in granzyme K due to newly identified pathogenic roles in aging, rheumatoid arthritis, airway inflammation, and skin injury and repair. Emphasis has been on the identification of pro-inflammatory roles of granzyme K, with the latest data supporting granzyme K to operate extracellularly to induce PAR signaling, activate complement, trigger pro-inflammatory cytokine release, enhance immune cell recruitment, and exacerbate the immune response to bacterial infections. The outcomes from these studies collectively support an emerging understanding of the role/s for granzyme K in disease and identify this protease as a potential therapeutic target.
